# Impact of treatment heterogeneity on drug resistance and supply chain costs^☆^

**DOI:** 10.1016/j.seps.2013.04.001

**Published:** 2013-09

**Authors:** Eirini Spiliotopoulou, Maciej F. Boni, Prashant Yadav

**Affiliations:** a MIT-Zaragoza International Logistics Program, Zaragoza Logistics Center, Zaragoza 50197, Spain; b Oxford University Clinical Research Unit, Wellcome Trust Major Overseas Programme, Ho Chi Minh City, Vietnam; c Centre for Tropical Medicine, Nuffield Department of Clinical Medicine, University of Oxford, Oxford, UK; d William Davidson Institute, University of Michigan, Ann Arbor, USA; e Ross School of Business, University of Michigan, Ann Arbor, USA; f School of Public Health, University of Michigan, Ann Arbor, USA

**Keywords:** Treatment heterogeneity, Drug resistance, Drug assortment, Costs of variety, Healthcare supply chains

## Abstract

The efficacy of scarce drugs for many infectious diseases is threatened by the emergence and spread of resistance. Multiple studies show that available drugs should be used in a socially optimal way to contain drug resistance. This paper studies the tradeoff between risk of drug resistance and operational costs when using multiple drugs for a specific disease. Using a model for disease transmission and resistance spread, we show that treatment with multiple drugs, on a population level, results in better resistance-related health outcomes, but more interestingly, the marginal benefit decreases as the number of drugs used increases. We compare this benefit with the corresponding change in procurement and safety stock holding costs that result from higher drug variety in the supply chain. Using a large-scale simulation based on malaria transmission dynamics, we show that disease prevalence seems to be a less important factor when deciding the optimal width of drug assortment, compared to the duration of one episode of the disease and the price of the drug(s) used. Our analysis shows that under a wide variety of scenarios for disease prevalence and drug cost, it is optimal to simultaneously deploy multiple drugs in the population. If the drug price is high, large volume purchasing discounts are available, and disease prevalence is high, it may be optimal to use only one drug. Our model lends insights to policy makers into the socially optimal size of drug assortment for a given context.

## Highlights

• We study the issue of socially optimal width of drug assortment for an infectious disease.
• We study the tradeoff between risk of drug resistance and operational costs.
• Treatment with multiple drugs results in better resistance related health outcomes.
• The marginal benefit decreases as the number of drugs used increases.
• We compare this benefit with the change in procurement and holding costs.

## 1 Introduction

Resistance against existing drugs is an extremely serious problem that undermines effective health care for millions of people [32,33]. The regulation and use of available drugs as well as the behavior of individuals and institutions strongly influence the evolution of resistance. In the absence of suitable economic incentives, decision makers (such as governments, physicians, patients) fail to take into account the negative impact of their policies or use of drugs on the future effectiveness of these products [42]. Even if the emergence of resistance to drugs is a naturally occurring biological phenomenon, this process may be accelerated or delayed by many factors one of the most important being the drug treatment strategies and policies implemented in different countries [26]. Major investments in drug development can be undermined if therapies lose efficacy due to inappropriate drug use; inappropriate use anywhere can generate drug resistance with implications for patients everywhere. The history of drug-resistance evolution in malaria is an instructive example. In the 1950s, resistance to chloroquine – the most commonly used antimalarial at the time - emerged in several locations and spread globally over the next two decades [38,41]. This led to the widespread adoption of antifolate drugs, and resistance to these drugs emerged shortly therafter [38]. This pattern is on the verge of being repeated for artemisinin-based drugs, the current globally used first-line therapy against malaria [19,22,34]. The use of multiple first line treatments for malaria remains almost nonexistent, partially because of concerns about higher programmatic and supply chains costs. The lack of rigorous analysis comparing the benefits and operational costs of using multiple drugs is contributing to global policy inaction on this issue.

In response to concerns about the evolution of drug resistance and the global health threat that its spread poses, many mathematical modeling studies have tried to understand the impact of using multiple therapies simultaneously in a population on the emergence and spread of drug resistance [5–8,10]. These studies have shown that when more therapies (drugs) are used simultaneously in the population, the emergence of drug resistance is delayed and its evolution is slowed. This is mainly due to a) the decrease in short-term and long-term drug-specific selection pressure resulting from the high number of available drugs and b) the slower degradation of the mean fitness of the parasite population, making it more difficult for new resistant types to invade and spread. In addition, the policy of using multiple drugs at the same time allows larger fraction of the population to be treated without trading off against future treatment of cases that may be untreatable due to high resistance levels.

While these studies show that increasing drug variety delays the emergence and spread of drug resistance, increased drug variety comes with additional operational costs. Volume discounts are a common practice in the pharmaceutical sector, implying that using more types of drugs for the same disease would result in a higher per unit price for the sourced drugs. Also, when demand is uncertain, wider drug variety for a specific disease implies higher safety stocks and therefore higher inventory holding costs. On the other hand, when all patients are treated with the same drug, there is a demand variability pooling effect that allows one to hold less safety stock in order to achieve the same customer (patient) service level (probability of stock-out during replenishment). For a given number of sourced treatments, increased drug variety leads to higher cost of procuring the drugs, increased inventory holding costs and in some cases may lead to increased healthcare worker training costs. This paper studies the tradeoff between risk of drug resistance and operational costs when using multiple drugs for a specific disease.

In this paper, we quantify the benefit of delayed resistance and disease containment associated with drug variety and compare it against the cost of higher variety in the supply chain. Our model lends insights to policy makers into the socially optimal size of the drug assortment. The rest of the paper is organized as follows: in section 2, we review the relevant literature in pharmaceutical economics and policy, epidemiology and supply chain management. In section 3, we employ a disease model that includes the emergence and evolution of resistance, we define disease burden using the concept of (Disability Adjusted Life Years) DALY, and by performing simulation analysis we show that disease prevalence and total resistance both decrease, in cumulative terms, with the number of drugs used. In section 4, we study together the directional effects of a wider drug assortment with respect to disease and resistance containment and procurement and safety stock holding costs. We complement the analysis by an extensive numerical study in section 5. In section 6, we summarize our results and present some policy insights regarding the socially optimal width of drug assortment.

## 2 Related literature

The field of pharmacoeconomics is concerned with optimal drug use. Issues of cost effectiveness are increasingly being placed alongside issues of clinical effectiveness in determining the desirability of introducing new drugs, especially given the rapid growth in drug expenditures combined with the ever increasing pressure to control healthcare expenditure [40]. Walley et al. [40] present methods of economic evaluation in relation to medicines [36], employs an economic framework to examine separately the supply and demand sides of the pharmaceutical market and [39] examines health public policy from a theoretical perspective employing economic theory.

Most of the literature that currently exists on the subject of antibiotic resistance is largely within the biological and medical science literature; optimal drug use has been addressed within an economic context by only a few researchers. Among the most notable papers that have dealt with the economic considerations surrounding biological resistance have been those of [11,25]. The first uses a very general analytical framework about both agricultural and human drug use, giving more attention to the private versus public aspect of the problem. The latter is among the first to recast an epidemiological model of antibiotic-resistant disease within an economic framework that considers the economic costs and benefits of treatment. Laxminarayan and Weitzman [27] consider the problem of optimal drug combination, recognizing the resistance problem associated with the uniform use of a single drug. They argue that a policy of uniform treatment of all patients based on the standard cost-effectiveness criterion may be inappropriate when drug resistance is endogenous, and selection pressure imposed by the use of any single drug leads sooner or later to the evolution of resistance to that drug. They consider resistance an externality that should be taken into account when socially optimal drug combination policy is considered. They show that a mixed treatment policy of multiple drug use is generally desirable. They assume that all patients receive treatment, effectiveness of all drugs is identical but their prices vary considerably.

Laxminarayan [23] attempts to bring together a variety of approaches to the economics of resistance by assembling papers in this emerging field of study. The book focuses on the use of economic tools to characterize the efficient use of antibiotics in the face of resistance as well as on the economic impact of resistance and decision making under uncertainty about future resistance. It also discusses how regulatory incentives might be structured for the pharmaceutical industry. Besides the theoretical and empirical work about drug resistance, its development, magnitude, key drivers and policy considerations [28]; CGDs [2,17], there has been some efforts to model the evolution of resistance and its effects in various contexts.

In the epidemiology literature, a number of papers have shown that using a variety of drugs in a population has better drug-resistance outcomes than using a single drug. A key question in such studies has been to determine how multiple drugs should be deployed, whether as (1) combination therapy, (2) cycling the drugs on a fixed schedule, (3) cycling on an adaptive schedule, or (4) distributing all drugs simultaneously (“mixing”). The earliest attempt, known to us, that considered the deployment of multiple antimalarial drugs in a population was a simple mathematical model described by [15]. The authors compared combination therapy to cycling individual drugs, and showed that the relative benefits of either strategy depend on the parasite recombination rate and drug coverage in the population. Bonhoeffer et al. [8] analyzed a general two-drug resistance model and concluded that cycling strategies fared worse than mixing strategies or combination therapies, when considering the total number of future cases of disease prevented. Bergstrom et al. [6] show that mixing drugs has an inherent advantage over cycling because mixing creates a more variable environment where drug-resistance evolution is less favored and slower for the disease in question. Smith et al. [37] show that a strategy of using multiple drugs can delay or even prevent drug-resistance evolution, whether the drugs are distributed by location, or whether multiple drugs are used in each location. However, this balanced system is sensitive to non-compliance; if spatial coupling is strong and if one location chooses a more selfish strategy, this can have adverse effects on other locations. Boni et al. [10] show that cycling regimes create a resistance-friendly environment earlier than mixing regimes; this study focuses on malaria transmission and shows through extensive numerical simulations that using multiple first-line therapies (MFT) simultaneously outperforms many types of cycling strategies.

## 3 Disease transmission and resistance evolution

While treatment homogeneity is valued by health policy experts and limited drug choice has many operational advantages, such a policy may be undesirable when drug resistance is endogenous. In this paper we consider the case of infectious diseases that are curable by some drug or therapy. Selection pressure imposed by the use of any single drug sooner or later leads to the evolution of resistance to that drug. Two social externalities inherently characterize the treatment of infectious diseases. On the one positive side, treatment cures the patient, thereby preventing the disease from being transmitted to other individuals. On the negative side, drug treatment selects in favor of mutations that confer drug resistance, increasing the likelihood that the drug will be less effective in the future. The individual patient fails to consider either of these externalities when deciding whether or not to receive a treatment.

### 3.1 Disease transmission model

A simple disease transmission model that includes compartments for susceptible, infected with resistant strain or drug-susceptible strain, can be used to understand how drug variety impacts resistance and disease burden. A basic model for a single drug, two and three drugs is presented in Appendix. Despite the parsimony of this disease transmission and resistance evolution model, for cases involving more than 3 drugs the number of differential equations required to define the system becomes very large. The number of equations increases exponentially in the number of drugs employed simultaneously in the population.

It is not possible to derive an analytical expression of disease prevalence and total resistance as a function of the number of drugs *n* used to treat the disease. The reason why is that the system of equations needed to describe the dynamics of the disease and to derive the analytical expressions of interest changes with the number of available drugs. We therefore proceed with building a simulation model and rely on numerical solution of such an ODE system to approximate the functions of interest, under various disease settings.

In order to incorporate more epidemiological realism into the analysis, we employ the malaria transmission model described by [10] (equations (A1)–(A5) in supplementary materials of [10]). This model is an ODE system that scales with size depending on the number of drugs being used; in this analysis we consider a maximum of ten drugs. The model accounts for immunity to malaria, allowing slow waning and acquisition of immunity for individuals that are infected a long time. Hosts can be in symptomatic or asymptomatic states, with only symptomatic hosts receiving treatment. The model tracks the circulation of all drug resistant and multi-drug resistant types to the ten drugs in the model, 1024 in total. Strains resistant to a single drug have a 1% fitness cost of resistance, and an additional 1% fitness cost is imposed for resistance to each additional drug. The multi-drug resistant strain with resistance to all ten drugs is 9.6% less fit than the sensitive wildtype strain.

We calibrated the model to four endemicity scenarios, with equilibrium prevalences set to 0.1% (very low), 0.5% (low), 5.0% (moderate), 25% (high). Equilibrium prevalences are obtained by determining the prevalence at endemic equilibrium when 50% of the population is treated with an effective drug, in the absence of resistance evolution. Because our calculated prevalences are not age-specific, they do not have an exact correspondence to the classical epidemiological classifications of malaria as hypoendemic, mesoendemic, hyperendemic, or holoendemic.

### 3.2 Disease burden and cost of resistance

For assessing the resistance burden under each scenario and therefore quantifying the cost associated with it, disability-adjusted life years (DALYs) are used [30,31]. The DALY is a widely used measure of overall disease burden in the field of public health and health impact assessment and combines the time lived with disability and the time lost due to premature mortality. It is defined as DALY = YLL + YLD, where YLL denotes the years of life lost due to premature mortality and YLD the years lived with disability [43]. The term “disability” is used broadly to refer to departures from good or ideal health in any of the important domains of health, following the standard terminology used to define global burden of disease. Social preferences for the point in time or age at which a death or disability occurs can be incorporated into DALY calculations, but for simplicity reasons we will assume that a healthy year now is equally preferred to a healthy year in the future (no discounting health with time) and that the value of each year of life does not depend on age (no social weighting).

In our context, we do not consider different death rates between infected population with a susceptible strain and infected with a strain resistant to a drug. Therefore disease and resistance burden are captured through the differences under each treatment scenario of disease prevalence and of treatment failures due to resistance emergence and spread.

Prevalence (*PR*) is defined as the total fraction of the population that is infected with any type of strain. Let *n* be the number of drugs employed simultaneously in the population, *W* denote the fraction of the population that is infected with a susceptible strain and *X*_*k*_ the fraction of the population that is infected with a strain resistant to drug or drug combination *k*. Then prevalence at a fixed time *t* is given by:

$$PR\left(n\right)=\sum_{k\in T}X_{k}+W$$

where *T* is the set of all subsets of *n*. We define total resistance (*TR*) at a given time *t* as:

$$TR\left(n\right)=\sum_{i=1}^{n}\frac{i}{n}g_{i}$$

where *g*_*i*_ is the fraction of all infected individuals that are infected with a strain resistant to exactly *i* drugs. Total resistance is a number between zero and one, and it is the probability that, a randomly chosen parasite would be resistant to a randomly chosen drug. Let *f*_*i*_ be the fraction of patients treated with drug *i*, *i* = 1,2,…,*n*. The Number of Treatment Failures (*NTF*) due to resistance are given by the expression:

$$NTF\left(n\right)=PR\left(n\right)\cdot TR\left(n\right)\cdot \sum_{i=1}^{n}f_{i}$$

Therefore, Disease Burden (*DB*) up to time *t* (time when the policy is evaluated) is given by the expression:

$$DB\left(n,t\right)=\int_{0}^{t}PR\left(n,t\right)\cdot \left[\left(1-\sum_{i=1}^{n}f_{i}\right)+TR\left(n,t\right)\cdot \sum_{i=1}^{n}f_{i}\right]\cdot D\cdot S\mathrm{d}t$$

where *D* is the average time of being sick when the patient receives no treatment or the treatment fails and *S* is a weight factor that reflects the severity of the disease into consideration. Note that the units of the disease burden are DALYs lost. By defining *V* as the value of one lost year of “healthy” life, usually mentioned in the literature as the value of a life year (VLY), we can define the Cost of Disease Burden: *CDB*(*n*,*t*) = DALYs ·*V*.

We proceed with approximating the functions of interest, under various disease settings, based on the results of our simulation model.

### 3.3 Numerical approximation of disease burden

Disease burden can be decomposed in two main parts: the burden due to infections with drug resistant organisms being treated with ineffective drugs (“resistance burden”) and the burden due to the individuals not receiving treatment at all (“untreated burden”). The impact of drug resistance is captured through the cumulative number of treatment failures up to time *t*, when the policy is evaluated, while the impact of the disease progress and the untreated burden through the cumulative prevalence of the disease. We ran simulations for different endemicity settings in order to estimate these quantities as a function of *n*, at different time points.

**Fig. 1:**
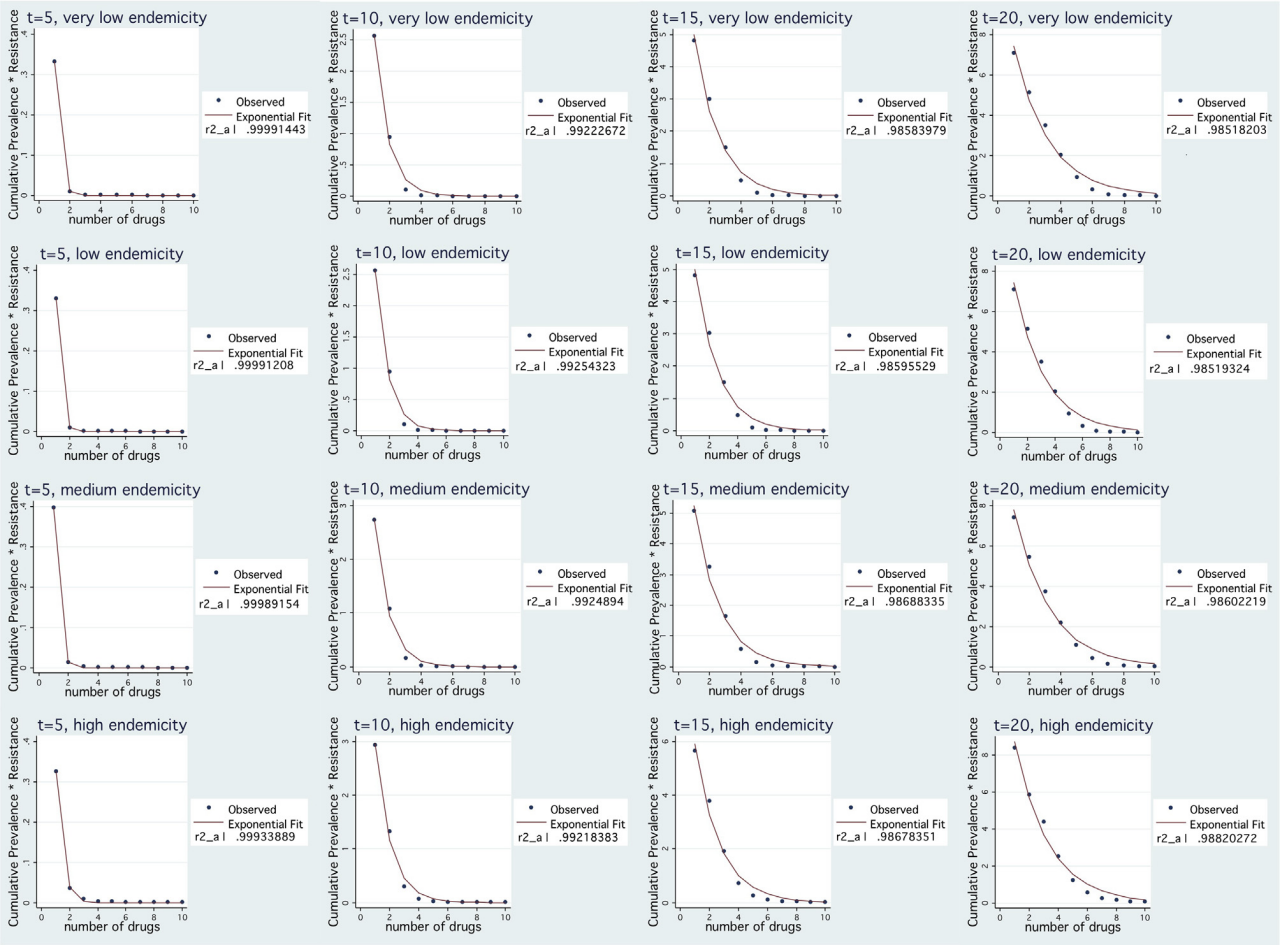
Exponential fit of NTF for a given t under various endemicity settings.

*Resistance Burden.* Simulation results for all endemicity settings, suggest that the *NTF*, evaluated at time *t* = 5, *t* = 10, *t* = 15 and *t* = 20, is decreasing exponentially with the number of available drugs. In all cases, for fixed *t*, $\int_{0}^{t}PR\left(n,t\right)\cdot TR\left(n,t\right)\mathrm{d}t$ can be very well approximated by an exponential function of *n* (using Stata/SE v10.0, goodness of fit - *R*^2^ - is higher than 0.98 in all cases). By fitting the simulation results, as shown in Fig. 1, we get:

$$\int_{0}^{t^{\text{*}}}PR\left(n,t\right)\cdot TR\left(n,t\right)\mathrm{d}t≃b_{0}e^{b_{1}n}$$

where *b*_0_ > 0 and *b*_1_ < 0.

**Fig. 2:**
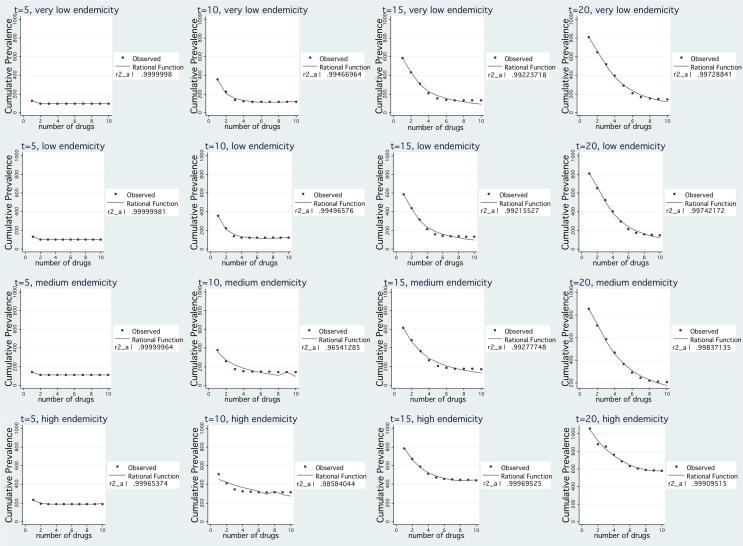
Rational function fit of cumulative prevalence for a given t under various endemicity settings.

*Untreated burden.* For given t, simulation results of cumulative prevalence of the disease suggest that it is a monotonically decreasing and convex function in *n*. Thus, cumulative prevalence can be well approximated well by rational function of *n* [35]. Fig. 2 presents the simulation and curve fitting results for a second order rational function. The approximation is very good (goodness of fit - *R*^2^ - is higher than 0.96 in all cases) and therefore we have evidence that:

$$\int_{0}^{t^{\text{*}}}PR\left(n,t\right)dt≃\frac{\beta_{0}+\beta_{1}n}{1+\beta_{2}n+\beta_{3}n^{2}}$$

where *β*_*i*_ (*i* = 0,1,2,3) are constants that depend on both the point in time when cumulative prevalence is evaluated and the endemicity setting.

Both resistance burden and disease prevalence are monotonically decreasing functions in *n* and consequently total disease burden decreases with drug variety. On the other hand, a wider drug assortment has cost implications on the supply chain; this is the topic of the next section.

## 4 Directional effects of higher variety in the supply chain

In this section we argue that for a given service level the number of available drugs determines the procurement costs and also safety stock holding costs. More specifically, we show that for a fixed number of procured treatments, higher drug variety leads to an increase in purchasing and holding costs. When we incorporate the effect of a wider drug assortment on disease prevalence and resistance containment, which determine the quantity of treatments needed in future periods, higher drug variety does not necessarily lead to an increase in purchasing and holding costs.

**Fig. 3:**
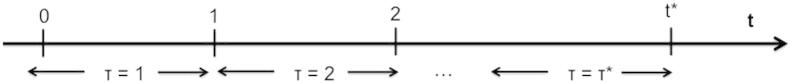
Time units.

We begin by modeling procurement and safety stock holding costs for one period under the assumption that demand and procured quantity is exogenous and fixed. We define a period as the time interval during which there is only one procurement opportunity. Then, we consider the multi-period case, where we incorporate the effect of number of drugs employed on future demand for treatments. While the disease transmission model is a continuous time model, operational costs are incurred per period (i.e. a cycle), in discrete time. The following schematic figure shows the correspondence (Fig. 3):

### 4.1 Procurement cost per period

Volume discounts based on the total quantity purchased over a given period are common in many industries, especially in business-to-business transactions. In the pharmaceutical sector, volume discounts are a common practice while in some cases national systems require price reductions if volume exceeds target levels [16]. We consider the case of a linear discount dependent on the total volume purchased applied to all units purchased.

Considering all unit quantity discounts, we model the unit price of a drug *i* as a decreasing function of the quantity purchased *q*_*i*_ per period of time, i.e. *c*_*i*_(*q*_*i*_) = *c*_0_−*kq*_*i*_, where *c*_0_ and *k* are positive constants and *c*_0_ > *kQ*, where *Q* is the total quantity procured per period for treatments for a specific disease in a locus. Denoting by *n* the number of available drugs for the same disease, *total procurement cost per period* (*PC*) is defined by: $PC\left(n\right)={\text{Σ}}_{i=1}^{n}\left[F_{i}+c_{i}\left(n\right)q_{i}\left(n\right)\right]$, where *F*_*i*_ denotes the fixed transaction cost per period for retaining a relationship with a supplier and ${\text{Σ}}_{i=1}^{n}q_{i}\left(n\right)=Q$. Assuming that all available drugs are equally priced for the same volume levels or that the least expensive ones (i.e. generics) are preferred, it follows immediately that when the unit price is a decreasing function of the quantity purchased and moreover there is a fixed transaction cost per available drug per period, procurement cost is increasing with the number of available drugs. In the special case where all available drugs are used in equal proportions, the total procurement cost function for a period can be re-written as $PC\left(n\right)={\text{Σ}}_{i=1}^{n}[F_{i}+c_{i}\left(Q/n\right)\left(Q/n)\right]={\text{Σ}}_{i=1}^{n}F_{i}+\left(c_{0}-k(Q/n)\right)(Q/n)=nF+\left(c_{0}-k(Q/n)\right)Q$.

### 4.2 Safety stock holding cost per period

Ensuring high product availability is of crucial importance in the health sector. In the presence of supply and demand variability, safety inventory is carried to satisfy any demand that exceeds the forecasted needs. Raising the level of safety inventory though comes at a cost; it increases inventory holding costs and the risk of product obsolescence, since shelf lives of drugs are often quite short.

Demand volatility for each drug increases with drug variety because demand for each drug depends not only on the real population need for treatment but also on other factors like patients' changing tastes or promotional efforts of pharmaceutical companies. Forecasting accuracy decreases with higher drug variety, as a result of lower demand aggregation. It is more difficult to forecast the demand for each individual drug than forecasting the need for treatments in total for a certain disease. For any product, the narrower the assortment, the higher is the pooling of the demand variability and the lower the total safety inventory required, when the customer service level is above 50% ([14]; p.318). Standard inventory theory states that a narrower product assortment would lead to lower inventory due to the benefit of risk pooling as long as demands across the different products are not perfectly positively correlated. Eppen [20] with his seminal work showed that total holding and stockout costs are lower when demand is aggregated, for independent and normal demand distributions and identical cost parameters (one-period setting). Eppen and Schrage [21] extend the result for the multiple period problem while [13] generalize Eppen's model to more general multivariate dependent demand distributions.

For the sake of simplicity, we assume that total demand for a given period is normally distributed with mean *D* and variance *σ*^2^, available drugs *n* are used in equal proportions and there is no demand correlation for drugs. The directional effects described by our model and the insights we get do not depend on these assumptions. In this case, the demand per period for each drug *i* follows the normal distribution; $N∼\left(D/n,\sigma^{2}/n\right)$. The required total safety stock, as a function of *n*, is: $SS\left(n\right)=nF^{-1}\left(CSL\right)\sigma_{L}/\sqrt{n}$, where *F*^−1^ is the inverse of the standard normal distribution, *σ*_*L*_ is the total demand standard deviation during the lead time and CSL the target service level (probability of stockout during the replenishment period) [14]. *Total safety inventory holding cost per period* is $HC\left(n\right)=h\bar{c}F^{-1}\left(CSL\right)\sigma_{L}\sqrt{n}$, where *h* is the holding cost per period and $\bar{c}$ the average unit cost of employed drugs in the same period. As shown above, $\bar{c}$ is increasing in *n*. Also, $\sqrt{n}$ is a monotonically increasing function. Thus, the cost of holding safety inventory is increasing as drug assortment is becoming wider. Total inventory held may increase or decrease depending on the patient service level policy makers decide to provide. In the case of substitutability, individual demands of employed drugs will be negatively correlated. In this case, inventory pooling benefits will be even higher and the provided inventory holding cost expression will serve as an upper bound.

*Observation 1.* Supply chain cost, defined as the sum of procurement and safety stock holding cost, is a monotonically increasing concave function in the number of drugs employed simultaneously in the population for a given time period, when demand and procured quantity can be regarded as exogenous.

Proof:

By taking the first and second derivatives with respect to *n* we get:

$$\begin{array}{l} \frac{dPC\left(n\right)}{dn}=F+k{\left(\frac{Q}{n}\right)}^{2}>0\\ \frac{d^{2}PC\left(n\right)}{dn^{2}}=-\frac{2kQ^{2}}{n^{3}}<0 \end{array}$$

and,

$$\frac{dHC\left(n\right)}{dn}=hF^{-1}\left(CSL\right)\sigma_{L}\sqrt{n}{\bar{c}}^{\prime }\left(n\right)+hF^{-1}\left(CSL\right)\sigma_{L}{\bar{c}}^{\prime }\left(n\right)\frac{1}{2\sqrt{n}}>0$$

given that ${\bar{c}}^{\prime }\left(n\right)=-kq_{i}^{\prime }\left(n\right)=kQ/n^{2}>0$. The second order derivative is given by:

$$\frac{d^{2}HC\left(n\right)}{dn^{2}}=-\frac{\alpha^{\prime }}{4\sqrt{n^{3}}}\bar{c}\left(n\right)+\frac{\alpha^{\prime }}{\sqrt{n}}{\bar{c}}^{\prime }\left(n\right)+\alpha^{\prime }\sqrt{n}{\bar{c}}^{\prime \prime }\left(n\right)$$

where $\alpha^{\prime }=hF^{-1}\left(CSL\right)\sigma_{L}$. Substituting ${\bar{c}}^{\prime \prime }\left(n\right)=-2kQ/n^{3}$ and after some algebraic manipulation, we get:

$$\frac{d^{2}HC\left(n\right)}{dn^{2}}=-\frac{\alpha^{\prime }kQ}{\sqrt{n^{5}}}-\frac{\alpha^{\prime }\bar{c}\left(n\right)}{4\sqrt{n^{3}}}<0$$

Thus, both procurement and holding cost are monotonically increasing, strictly concave functions in *n*. By defining supply chain cost *SC*(*n*) as the sum of procurement and holding cost, we have: *dSC*(*n*)/*dn* > 0 and *d*^2^*SC*(*n*)/*dn*^2^ < 0. Supply chain cost, is an increasing concave function in *n*, as the sum of two monotonically increasing concave functions. Note that the definition of *PC*^′^(*n*) and *HC*^′^(*n*) holds for *n* > 0, as the procurement and holding costs functions, as defined in our model, are discontinuous at *n* = 0. Assuming *n* is continuous random variable, as we move from *n* = 0 to *n* = 0 + *ε* we treat from 0 a constant number of patients *Q*. In our analysis, we are focusing on the issue of the optimal number of drugs employed to treat a disease, and not on whether the disease should be treated or not.

### 4.3 Multi-period case with endogenous demand

In this section, we consider a multi-period setting where demand for treatment in future periods depends on the width of drug assortment employed in a given period. As before, we compare the benefits of delayed and slower resistance evolution from a wider drug assortment with operational costs, but the demand in future periods (or quantity of treatments procured) is not exogenous or fixed but depends on the decisions on drug variety made in each time period.

Simulation results suggest that disease burden, for a given *t*^∗^, can be expressed as:

$$DB\left(n\right)=\left[\frac{\beta_{0}+\beta_{1}\cdot n}{1+\beta_{2}\cdot n+\beta_{3}\cdot n^{2}}\left(1-\sum_{i=1}^{n}f_{i}\right)+b_{0}e^{b_{1}\cdot n}\sum_{i=1}^{n}f_{i}\right]\cdot D\cdot S$$

where *β*_0_,*β*_1_,*β*_2_,*β*_3_ are such that:

$$\frac{\beta_{1}\left(1+\beta_{2}n+\beta_{3}n^{2}\right)-\left(\beta_{0}+\beta_{1}n\right)\left(\beta_{2}+2\beta_{3}n\right)}{{\left(1+\beta_{2}n+\beta_{3}n^{2}\right)}^{2}}\leq 0$$

and *b*_0_ > 0, *b*_1_ < 0. The above expression is a monotonically decreasing convex function on *n*, as the weighted sum of two decreasing convex functions.

According to our definition, disease burden for a period *τ* is:

$$DB_{\tau }\left(n\right)=\int_{t-1}^{t}PR\left(n,t\right)\cdot \left[\left(1-\sum_{i=1}^{n}f_{i}\right)+TR\left(n,t\right)\cdot \sum_{i=1}^{n}f_{i}\right]\cdot D\cdot S\mathrm{d}t$$

We model the total demand for treatments in period *τ* as an increasing function of disease prevalence in period *τ*−1and the total procured quantity in period *τ*, *Q*_*τ*_, as an increasing function of demand of the same period. We have: *D*_*τ*_ = *H*(*PR*_*τ*−1_(*n*)), *Q*_*τ*_ = *G*(*D*_*τ*_) and $H^{\prime }\left(PR_{\tau -1}\left(n\right)\right)>0$, $G^{\prime }\left(D_{\tau }\right)>0$. Denoting by *τ*^∗^ the number of periods within *t*^∗^, total procurement cost is given by:

$$PC\left(n\right)=\sum_{\tau =1}^{\tau^{\text{*}}}\sum_{i=1}^{n}\left[F_{i}+c_{i,\tau }\left(n\right)q_{i,\tau }\left(n\right)\right]=\tau^{\text{*}}nF+\sum_{\tau =1}^{\tau^{\text{*}}}\left[\left(c_{0}-k\frac{Q_{\tau }}{n}\right)Q_{\tau }\right]$$

where the second equality holds for *n* identical drugs in terms of cost parameters and usage. It is straightforward to see that fixed costs go up as *n* increases. Variable costs though, may either increase or decrease as *n* increases. The total number of sourced treatments over time decreases $\left(Q_{\tau }^{\prime }\left(n\right)=(dG\left(D_{\tau }\right)/dD_{\tau })dH\left(DB_{\tau -1}\right)/dDB_{\tau -1})(dDB_{\tau -1}\left(n\right)/dn)<0\right)$. On the other hand, the price per unit of sourced treatment increases in *n* $\left(dc_{i,\tau }/dn=d/dn\left[c_{0}-kQ_{\tau }\left(n\right)/n\right]=(-nkQ^{\prime }\tau \left(n\right)+nkQ\tau \left(n)\right)/n^{2}>0\right)$.

The total safety stock holding cost for *τ*^∗^ periods is given by the expression:

$$HC\left(n\right)=\sum_{\tau =1}^{\tau^{\text{*}}}h{\bar{c}}_{\tau }\left(n\right)F^{-1}\left(CSL\right)\sigma_{L,\tau }\sqrt{n}=\sum_{\tau =1}^{\tau^{\text{*}}}\alpha {\bar{c}}_{\tau }\left(n\right)\sigma_{L,\tau }\sqrt{n}$$

where $\alpha =hF^{-1}\left(CSL\right)$ and ${\bar{c}}_{\tau }\left(n\right)$is the average unit cost in period *τ* of all drugs employed. It is reasonable to assume that standard deviation of demand during lead time is proportional to the mean demand of the same period (i.e. constant coefficient of variation). We therefore define $\sigma_{L,\tau }^{\prime }\left(D_{\tau }\right)=\gamma$, where *γ* > 0. We note that as the number of available drugs increases, both ${\bar{c}}_{\tau }\left(n\right)$ and $\sqrt{n}$ increase. The standard deviation of demand during the lead time, however, decreases with *n* as *D*_*τ*_ is a decreasing function of *n*. Therefore, depending on the specific parameters, safety stock holding costs may increase or decrease as drug variety becomes wider.

*Observation 2.* When demand is endogenous and the procured quantity of treatments per period is based on the level of disease transmission and resistance evolution dynamics, supply chain cost is not necessarily increasing on the number of available drugs.

It follows directly from the analysis above that while fixed procurement cost and the per unit price of the sourced treatments increases, the total quantity to be procured decreases as drug variety becomes wider due to the benefit of disease and resistance containment. Regarding safety stock, smaller drug variety on one hand favors demand variability pooling but on the other hand increases demand and thus its variability during the lead time. We note that the total relevant cost function, defined as the sum of disease burden and supply chain cost, may not be unimodal in the number of drugs employed simultaneously.

We show that for a given time horizon mixed treatment policies with multiple drugs result in a better health outcome when factoring in disease burden and resistance evolution. More interestingly this additional benefit is decreasing as the number of available drugs increases. Increasing drug variety may come at an operational cost though, increased procurement and holding costs. Thus, for certain range of disease transmission and cost parameters a clear trade-off will exist when deciding about the width of drug assortment; there is a point beyond which the additional health benefit and the corresponding decrease in quantity of treatments will not compensate for the increased operational costs from higher drug variety. Policy makers in resource limited settings need to explicitly consider this trade-off to achieve sustainable health outcomes over a longer time horizon. In the next section we conduct extensive numerical analysis to explore the endemicity settings, drug prices and volume discount parameters where this trade-off is most important to consider.

## 5 Numerical analysis

We begin our numerical analysis for a base population of one million and for *t* = 15 years. We use the international disability weight for malaria^1^ for severity factor and we consider 20 days (it is later varied from 5 to 30 days) as the average time of being sick when there is no successful treatment. As for the value of one year of healthy life lost, there are still unresolved issues for estimating it and the range of proposed values varies considerably among studies [1] and countries. We are using the value of $1000 per DALY implying a “health cost” of each untreated patient (ignoring externalities) of about $12 (same magnitude as the average cost of a treatment in the base case).

**Fig. 4:**
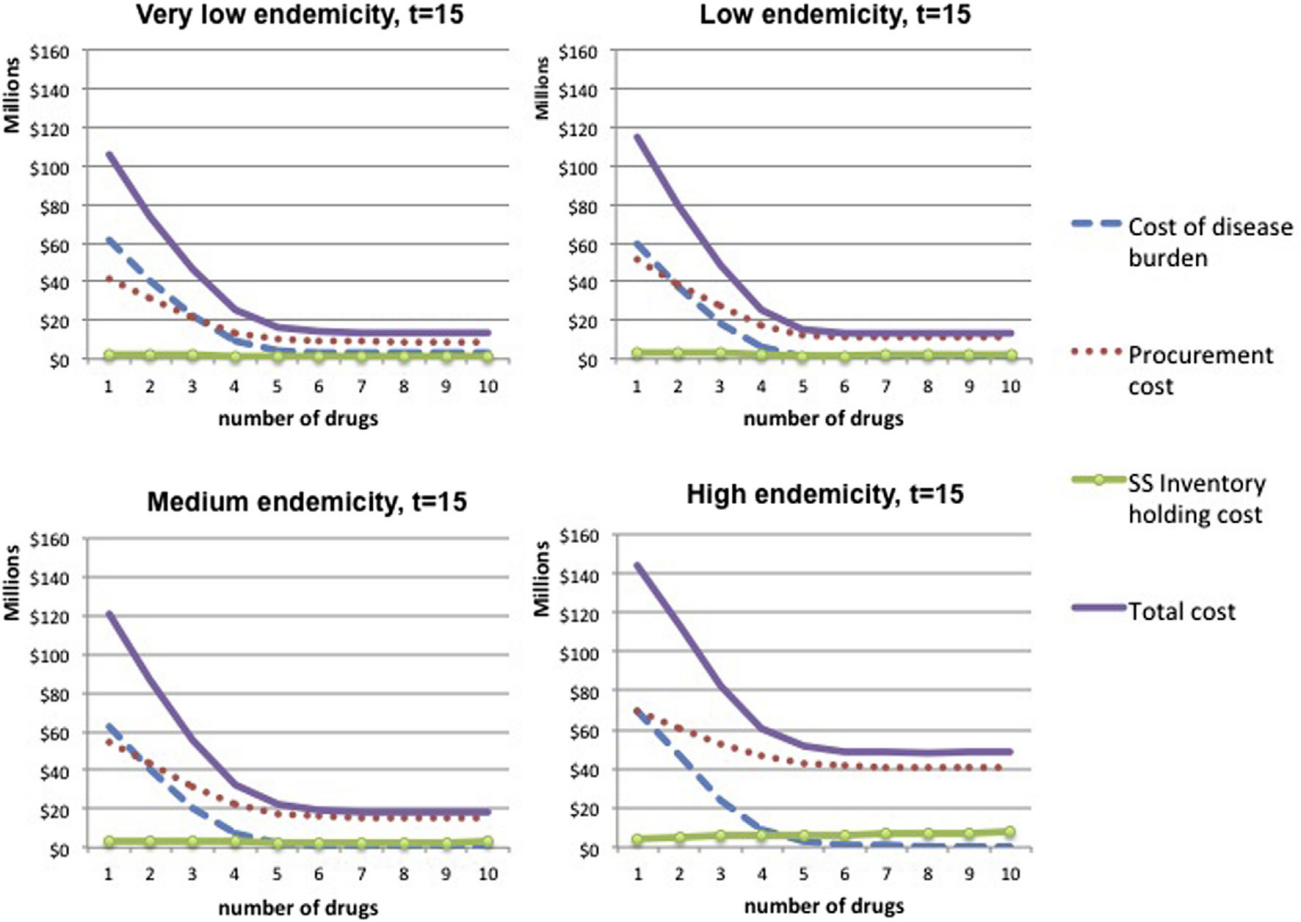
Relevant costs as a function of drugs employed for different endemicity settings, *p* = $10.

For an average drug price of $10 and a volume discount of 1% for 100,000 units, the total procurement cost is calculated. The fixed cost of ordering per supplier per period is set to $1000. The safety stock needed to absorb fluctuations in supply and demand is calculated using standard deviation of demand during the lead time equal to *μ*/4, where μ is the average demand per period. The probability of a drug stock-out during the lead time is set to 5% and an annual inventory holding cost of 15% is assumed. Fig. 4 shows the results for the very low, low, medium and high endemicity settings described in Section 3.1.

We observe that the total cost function, after the point where it reaches its minimum, remains quite flat as the number of drugs increases. As expected, for low values of *n*, the cost of disease burden decreases significantly in the number of drugs employed simultaneously due to the benefit of resistance containment and the decrease in disease prevalence. After a point though, it remains quite flat. This is because the marginal health benefit of an additional drug is heavily decreasing as the width of variety increases, as seen in Figs. 1 and 2. Procurement cost function behaves in a similar way mainly because the number of sourced treatments depends on disease prevalence. The rate at which procurement costs decrease as *n* increases is lower than the rate of disease burden decrease because higher variety leads to higher unit cost per sourced treatment and higher fixed costs. In accordance with the analysis in Section 4.2, safety stock holding cost keeps increasing as *n* increases, at a flat rate, but its magnitude is small compared to the other two cost components.

**Fig. 5:**
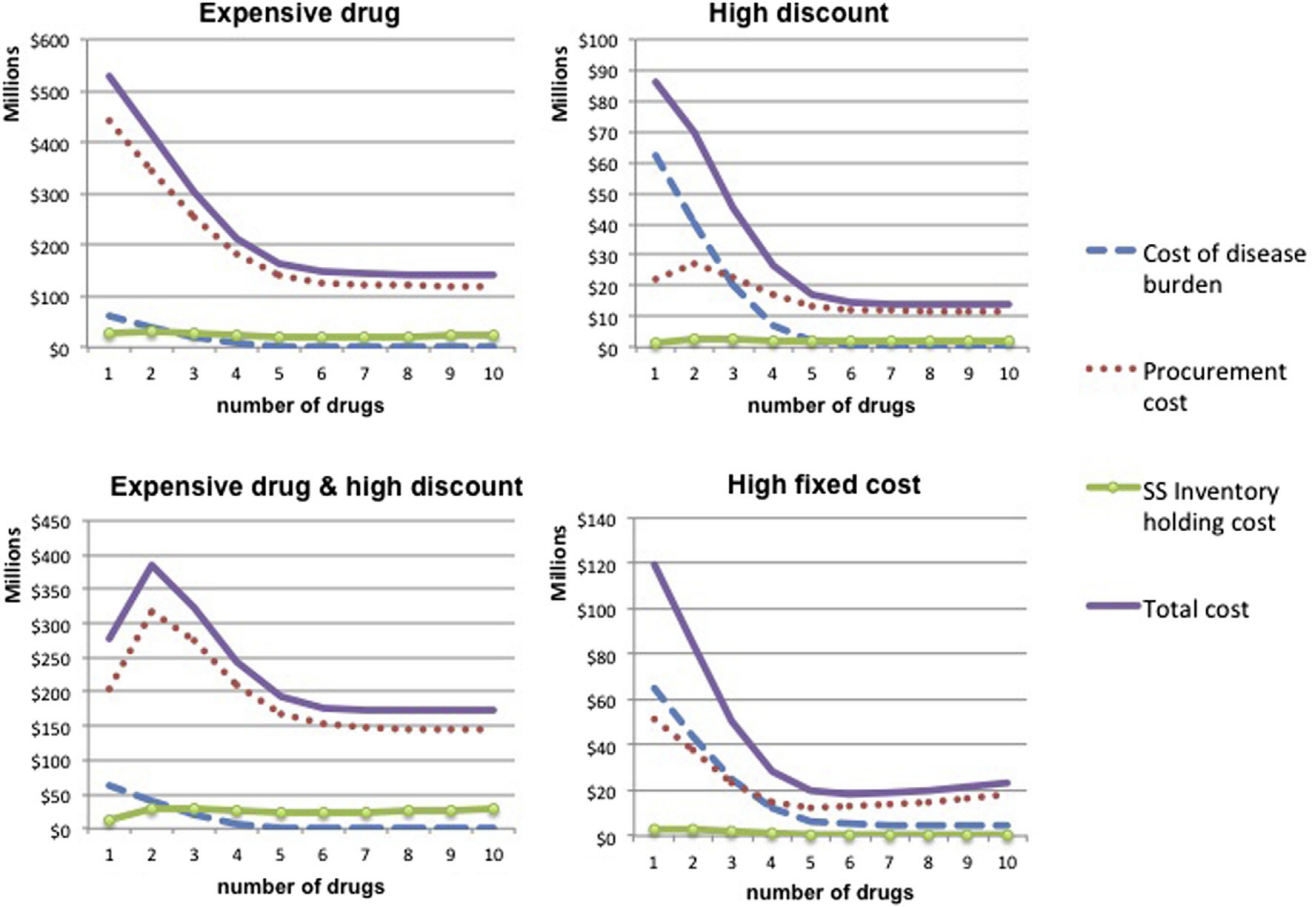
Medium endemicity: Relevant costs as a function of drugs employed for different drug cost parameters (price, volume discount, fixed cost).

From the analysis in Section 4, it becomes apparent that the behavior of supply chain costs depends heavily on the discount scheme and the fixed cost per supplier per period. For medium endemicity setting and *t* = 15, Fig. 5 presents how the various cost elements behave as a function of *n* when the price of the drug is high, when there are high volume discounts and when the fixed cost per supplier is very large.

**Fig. 6:**
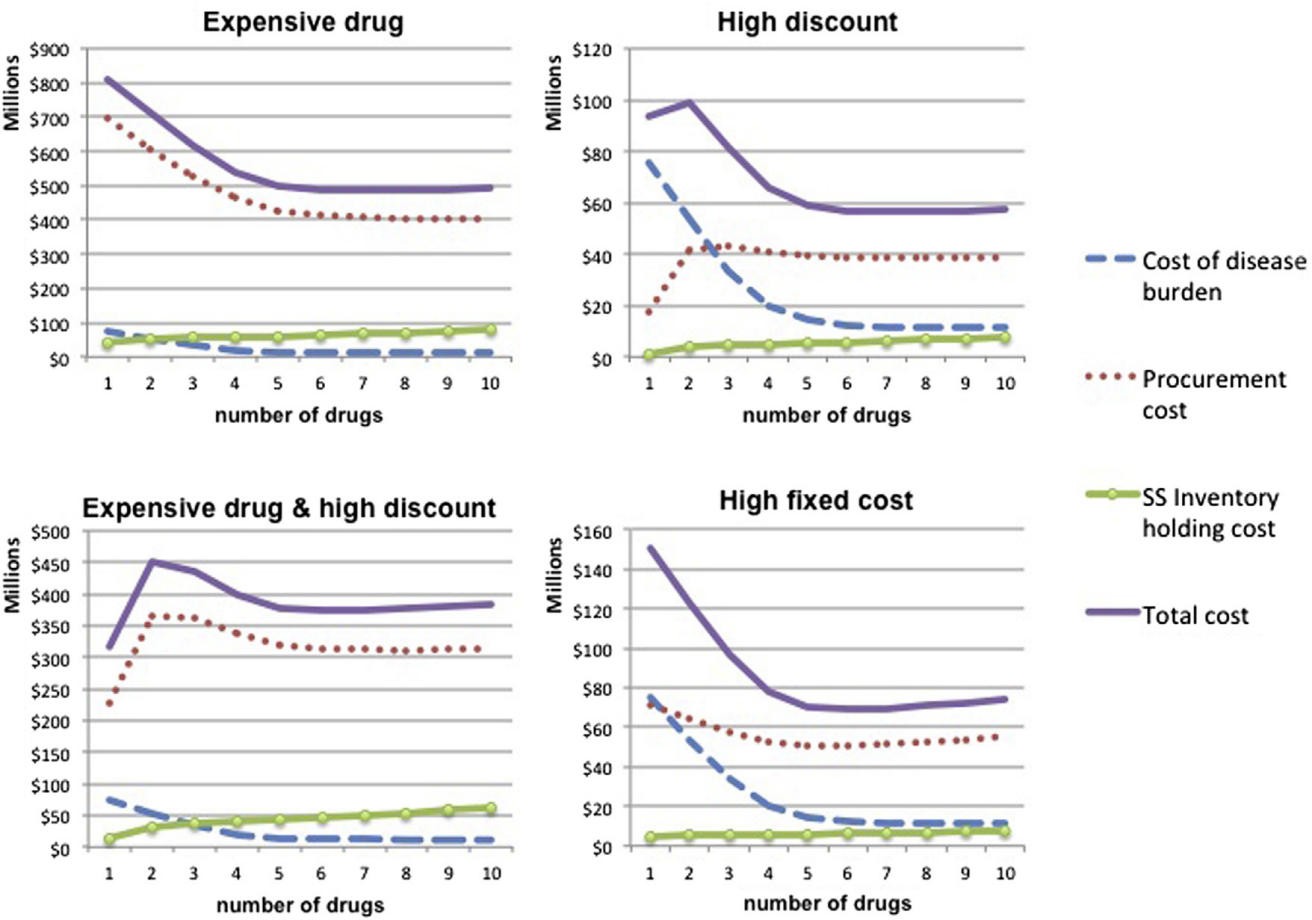
High endemicity: Relevant costs as a function of drugs employed for different drug cost parameters (price, volume discount, fixed cost).

When high volume discounts are applicable, procurement costs increase and then decrease as variety increases. For example, when we move from one to two different drugs, for a fixed number of treatments, the volume procured from each supplier reduces to half. The potential price decrease based on volume is lost and, if volume discount is high, it is not compensated by the fact that less treatments are procured in total (due to the health benefit associated with two compared to one drug). This trend is reversed as the number of *n* increases because the potential gains from volume discounts are decreasing. In the presence of very high fixed cost per sourced drug, procurement cost increases when the drug variety is large and *n* is increasing. For high values of *n*, the fixed costs, which increase linearly in *n*, dominate the additional benefit of sourcing less treatments (which is decreasing as *n* increases). As expected, when the price of the drug is high (compared to the health cost of non-treating a patient), procurement cost is the main driver of total cost. Similar behavior is observed for the other three endemicity settings, while these effects become more pronounced the longer the time horizon considered. For expositional purposes, we present also the analogous results for high endemicity setting (Fig. 6).

**Fig. 7:**
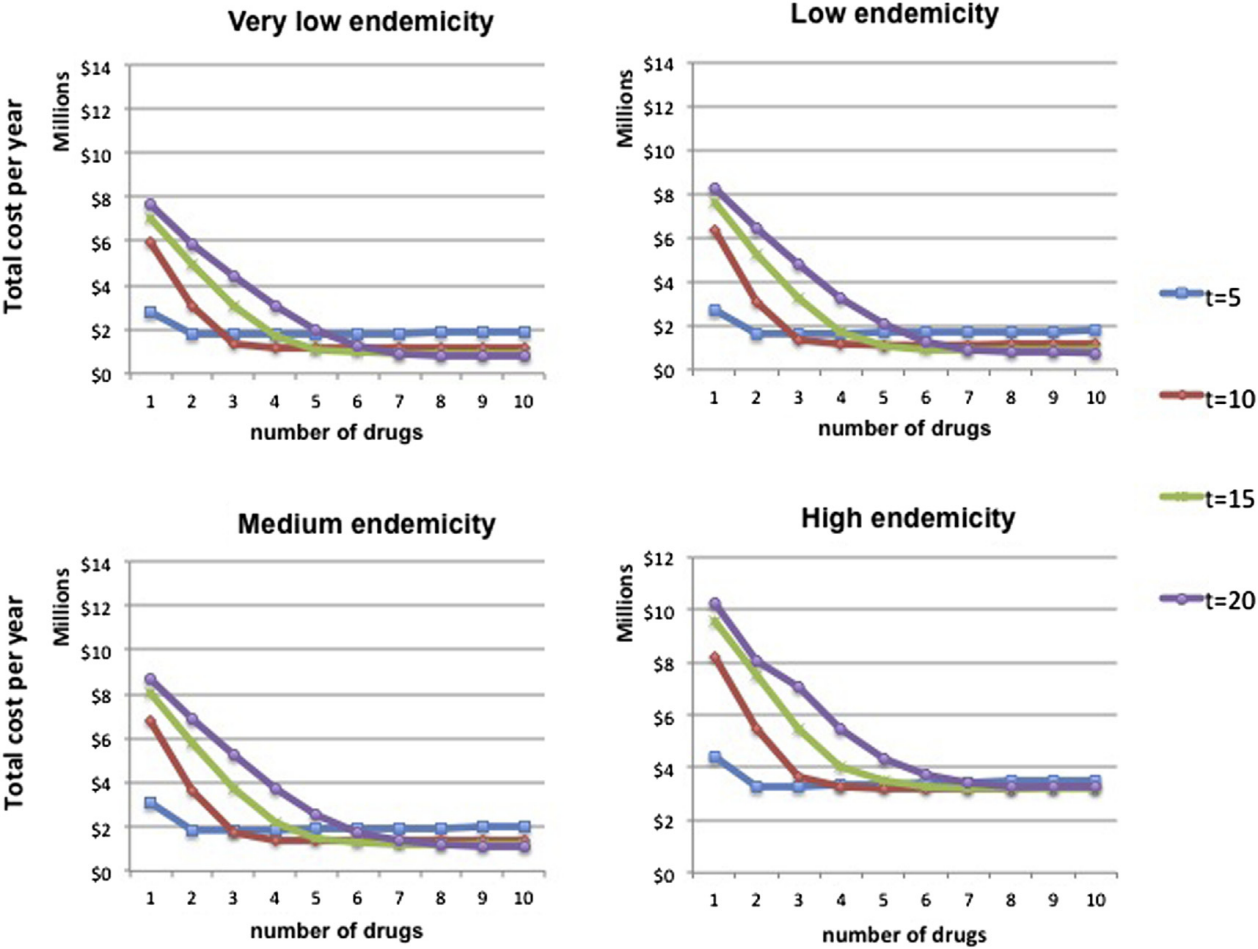
Average total cost per year when the time horizon varies.

The appropriate time horizon to be considered depends on the disease under consideration and the expected time to the development of next-generation drugs. All other things equal, we study the effect of the chosen time horizon on the optimal number of drugs to employ. For the base case described above, Fig. 7 shows, for each endemicity setting, how the average total cost per year function behaves when the policy is evaluated at different time points. In all cases, the longer the time horizon considered, the larger the width of the optimal drug assortment.

We continue by employing a full factorial design to assess the impact of the cost of the drug, the magnitude of the quantity discounts offered and the disease duration (by modifying the average time of being sick when treatment fails) on the optimal number of drugs. Table 1 summarizes the results of the analysis by indicating the optimal number of drugs for each case and for *t* = 10 years. (Table 2).

The results of the analysis are very similar under all four endemicity scenarios; in some cases the number of optimal drugs to be employed simultaneously is slightly higher in higher endemicity settings. Even if this may seem counterintuitive, it results from the fact that the shape of the disease burden curve as a function of the number of drugs *n* remains similar under all endemicity settings. Disease prevalence seems to be a less important factor when deciding about the width of drug assortment compared to the disease duration. The only exception is when both the price of the drug and the volume discount offered are very high. The results show that in such a case, in the high endemicity scenario, it may even be optimal to employ only one drug. This is not the case under any other lower endemicity scenario.

For low and medium drug prices, the duration of disease when there is no treatment or the treatment is ineffective, is a significant factor determining the optimal number of drugs. When the average price of the drug is as small as $1, supply chain costs are of secondary importance. Especially when the severity of the disease is high and endemicity is high, it is optimal to employ simultaneously all available drugs. On the other hand, in the examples where the drug price is $100, the optimal number of drugs depends less on the severity of the disease and it even remains unchanged in the medium endemicity setting. In such cases, procurement cost is the main driver of total cost; a wider variety contains procurement costs, up to a point, through the more effective disease containment that results in less sourced treatments in total.

Volume price discounts have an effect on the optimal number of drugs in the extreme case of high endemicity, high drug price and high discount. The results show that when the price of the drug is as high as $100, high volume discounts (that result in 10% price reduction for 100,000 units procured) may reduce the optimal number of drugs from 5 to 1 when disease endemicity is high. The magnitude of the discount mainly determines the shape of the procurement cost function (see Figs. 5 and 6).

**Fig. 8:**
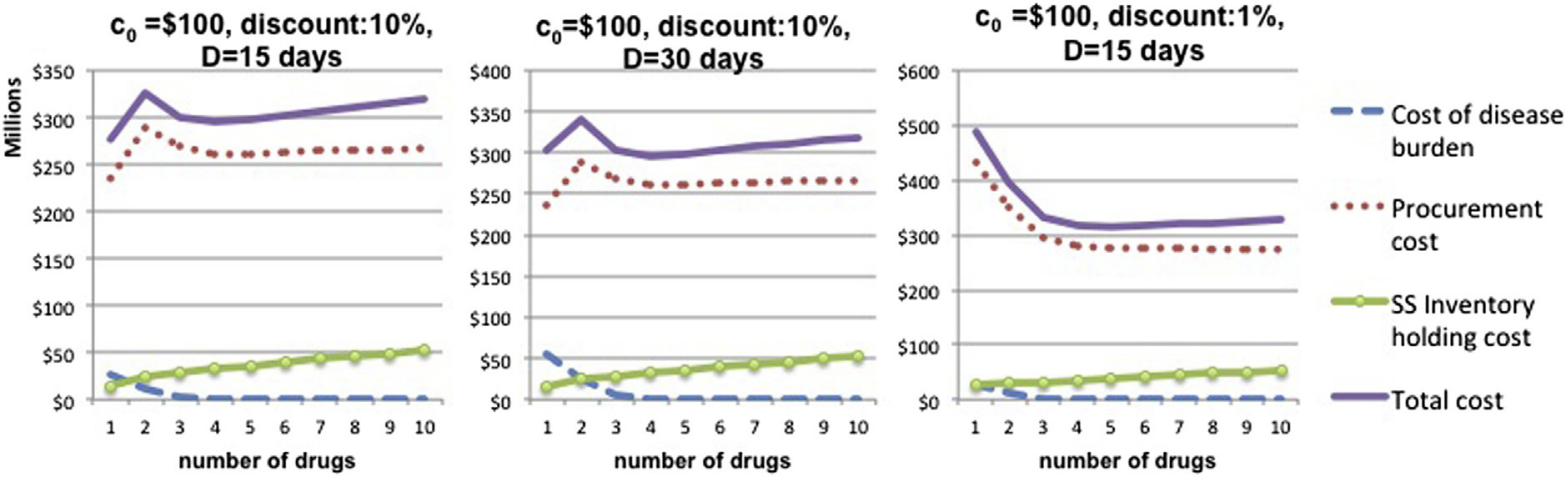
Cost breakdown and optimal *n* for high endemicity settings when the cost of the drug is high: the effect of volume discounts and disease severity.

We further explore this result - that it is optimal to employ only one drug - by studying the cost breakdown structure, as a function of *n*, for the case where the disease duration is 15 days (Fig. 8). We compare the relevant cost functions with the case where disease duration is increased to 30 days (optimal *n* in this case is 4) and the case where volume discount per 100,000 is reduced to 1% (optimal *n* is 5). We observe that for high discount, the total cost behavior is similar when both the disease duration is 15 and 30 days: total cost function has two local minima. The crucial difference is that for lower disease duration, even if total cost decreases for 2 < *n <* 4, its global minimum is attained at *n* = 1. When the disease severity is higher (i.e. disease duration is 30 days) the local minimum at *n* = 4 is also global minimum. On the contrary, when volume discount is lower, procurement cost, the main driver of total cost, is convex in *n*: monotonically decreases up to *n* = 5 and then starts increasing.

## 6 Discussion

Drug efficacy is a public good that is threatened by the emergence and spread of resistance. Available drugs for the treatment of curable diseases should be used in a socially optimal way, explicitly considering the negative externalities associated with use. Various studies have argued that when more than one drug is employed in a population at any given time, the emergence and spread of drug resistance is delayed. In this paper, we compare the benefits of resistance and disease containment that may result from using a wider drug assortment in a given population with the corresponding increases in operational costs such as procurement and inventory holding costs. We employ a simple general disease transmission model that allows for treatment with more than one drug to estimate the disease burden and cost of resistance. We proceed by building a simulation model in order to numerically approximate disease prevalence and total resistance, the two main components of disease burden, as a function of the number of drugs employed simultaneously. We show that disease burden (as defined in this paper) decreases with the number of drugs used. On the other hand, for a given level of demand and procured quantity, higher drug variety results in increased procurement cost when volume discounts are present, and in increased safety stock holding cost, when demand is uncertain. Drug variety at a given time also impacts future demand because of disease expansion and resistance evolution. When this effect of drug variety on future demand is considered, then higher drug variety does not necessarily lead to monotonically increasing procurement and safety stock holding cost.

Large scale numerical analysis shows that when the severity of the disease is high and the average price of the drugs is as small as $1, it is optimal to simultaneously employ all available drugs. In such cases supply chain related costs are very small as compared to the disease burden related costs and it is always better to use more drugs. This insight suggests that countries in sub-Saharan Africa with high malaria burden should consider incorporating as many available malaria medicines in their treatment guidelines and not worry about the higher supply chain costs resulting from using a wider assortment of drugs.

Only in some select instances when the disease endemicity is high, the drugs are expensive (>$100), and manufacturers offer significant volume purchasing discounts it would be optimal to use only one or very few of the available drugs. Infectious diseases with expensive drugs and high endemicity include HIV/AIDS and MDR-TB but the disease transmission and resistance development model used in this paper does not directly apply to those diseases.

In all cases, the optimal width of drug assortment decreases with the cost of the drugs. When drugs are cheaper it is optimal to use most or all of the available drugs. For cheaper drugs, the optimal variety increases with the disease duration. When drugs are cheap the cost of disease burden is the main driver of total cost and disease duration is an important component of the disease burden. For more expensive drugs and/or when higher volume discounts are available, the duration of the disease is of lesser importance. The main reason is that the most significant component of total cost in this case is the cost of procuring the drugs. Variable procurement cost may decrease with variety because the number of sourced treatments is reduced. On the contrary, the unit price and the fixed cost dealing with each supplier increase with the number of drugs employed simultaneously. Thus, when the additional health benefit of variety is small (around 5 drugs in our examples) another drug is not justified; it leads to an increased unit price and additional fixed costs not compensated with the decrease in the number of treatments sourced.

The numerical analysis also shows that compared to the duration of the disease and the price of the drugs, disease endemicity appears to be a less important factor in determining the optimal width of drug assortment. This implies that for a given disease e.g. malaria the optimal drug assortment will be the same or at least very similar across countries with varying levels of endemicity. This greatly simplifies the social planner's problem and implies that each country's optimal assortment problem does not need to be solved individually.

The model has several limitations. We assume that the diseases considered do not influence the patients' death rate and thus they do not affect population dynamics. This may not be the case for some infectious diseases with a very high associated mortality. Furthermore, overall demand for drugs for some disease may be affected by the assortment of drugs available. For given prevalence that determines the size of the market (an upper bound of demand), wider drug variety may lead to an increase of treatment seeking, according to consumer theory. An interesting extension and a topic of further research would be to incorporate the effect of drug assortment on the fraction of the patients that seek treatment.

Procurement cost is often not a linear function of quantity as is modeled in this paper. Hence, when this analysis is applied to a specific situation, we need to be aware what part of the procurement–cost curve we are working with. For example, at a national level, volume discounting may have a negligible effect if a country decides to purchase three million instead of two million doses of a drug. Similarly, supranational pooled purchasing arrangements may eliminate some of the volume discount effects.

In this paper we assume that all available drugs are used in equal proportions. However, it remains unclear what treatment guidelines to healthcare providers can achieve this equal and uniform use of multiple drugs. In addition, we need to be able to determine if moderate deviations from a uniform distribution of drugs have small or large effects on procurement costs, holding costs, and disease burden. Boni et al. [10] show that modest deviations from uniformity should not lead to large increases in disease burden. Also, we do not consider the costs associated with additional healthcare worker training as a result of using a treatment protocol involving multiple drugs. Although few estimates are available for this cost at present, the cost of training health personnel could be significant.

Finally, in many situations the number of drugs available to treat a disease is very limited to start with. For instance, a country managing its antimalarial supply would realistically only consider the simultaneous use of at most two or three drugs, given various logistical challenges and the fact that only certain antimalarials (artemisinin-combination therapies) are the most likely ones to be used due to their high cure rates. The analysis conducted in this paper may appear less relevant for such settings today but has high relevance for policy makers as they think about future drug assortments.

## Figures and Tables

**Fig. 9:**
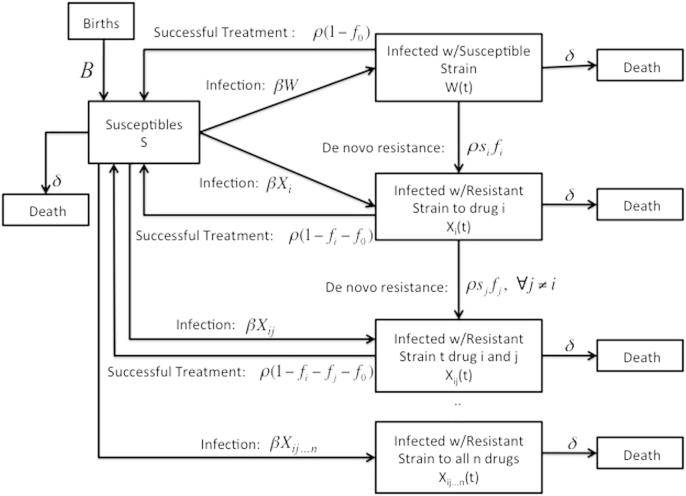
Disease transmission and resistance evolution in case of *n* drugs.

**Table 1:** Optimal number of drugs: Left-hand column shows disease duration. Top row in each table is the price of the drug; second row is the quantity discount.

*	$1			$10			$100		
	0.1%	1.0%	10%	0.1%	1%	10%	0.1%	1%	10%
Very low endemicity									
5 days	5	5	5	5	5	5	5	5	4
15 days	6	6	6	5	5	5	5	5	4
30 days	7	7	6	5	5	5	5	5	5
60 days	8	8	8	6	6	5	5	5	5
Low endemicity									
5 days	5	5	5	5	5	5	5	5	4
15 days	6	6	6	5	5	5	5	5	4
30 days	7	7	6	5	5	5	5	5	5
60 days	8	8	8	6	6	5	5	5	5
Medium endemicity									
5 days	6	6	5	5	5	5	5	5	5
15 days	7	7	6	6	6	5	5	5	5
30 days	7	7	7	6	6	5	5	5	5
60 days	9	9	8	6	6	6	5	5	5
High endemicity									
5 days	6	6	5	5	5	5	5	5	1
15 days	7	7	7	5	5	5	5	5	1
30 days	9	9	8	6	6	5	5	5	4
60 days	> = 10	> = 10	> = 10	6	6	6	5	5	4

*All unit volume discount that result in 0.1%, 1% and 10% unit price reduction for 100,000 units bought.

**Table 2:** Summary of notation.

Symbol	Definition
B	Birth rate
*β*	Transmission rate constant
*s*_*i*_	Probability that de novo resistance emerges when drug *i* is used
*ρ*	Rate at which treated individuals return to susceptible state
*δ*	Death rate
*S*	Susceptible population
*W*	Population infected with a susceptible strain
*X*_*i*_	Population infected with a strain that is resistant to drug *i*
*I*	Infected population
*f*_*i*_	Fraction of patients (symptomatic) who are treated with drug *i*, *i* = 1,2,..*n*
*f*_0_	Fraction of patients who are treated with no drug
*n*	Number of drugs

## Appendix

### Simple disease transmission model

To take into consideration the externalities associated with the treatment of infectious diseases and assess how disease spreads and resistance emerges under various drug deployment policies, we extend the simplest general disease model (SIS) [3,18] that allows for treatment with more than one different drug to include the emergence and evolution of resistance to a drug (for a similar models see [12,24]).

#### Assumptions

The main underlying assumptions of this model are the following:

• Mass-action mixing. The contact rate between susceptible individuals and infected individuals is proportional to the product of their respective densities.
• The transmission rate β is constant in time. This parameter incorporates the expected number of contacts per unit of time per individual and the probability of disease transmission.
• An infected individual becomes immediately infectious.
• No immunity. We consider here a disease that confers no immunity, or a very weak level of immunity on its host (in Section 3.1 we consider a malaria model where individuals can have partial immunity to malaria).
• No fitness cost of resistance. Typically, drug-resistant organisms have a lower evolutionary fitness than their drug-sensitive counterparts, which can manifest itself as slightly slower reproduction or quicker death [4,9]. In this version of the model, we do not account for the fitness cost that resistance imposes generally on the parasite.
• The susceptible population is constant. Most disease transmission models have an endemic equilibrium state where the birth of new susceptible is balanced by the loss of susceptible to infection and death. For simplicity, in this section, we assume that the susceptible population is constant.

This model for disease transmission and resistance evolution when *n* drugs are employed simultaneously is presented schematically in Fig. 9.

Population is divided into Susceptibles (*S*) and Infected with a either a susceptible strain (*W*) or with a strain that is resistant to a drug *i* (*X*_*i*_) or drug combinations (*X*_*ij*..*n*_). The fraction of patients, all symptomatic, that is treated with drug *i* is denoted by *f*_*i*_ and *f*_0_ refers to the fraction of the infected population that does not receive any treatment. By definition, $\sum_{i=0}^{n}f_{i}=1$. The rate at which treated individuals return to susceptible state is *ρ*. Infected and susceptibles die at a rate *δ* and deaths are balanced by births into the uninfected population (*B* denotes the birth rate).

A susceptible individual may either get infected by a drug-sensitive strain or a strain resistant to any drug or drug combination at a rate β, which refers to the transmission rate constant, *times* the fraction of population infected with the type of strain under consideration. Infected patients that receive treatment return to the susceptible state at a rate *ρ* provided that they are treated with an effective drug. De novo resistance arises when an infected patient is treated with a drug; the rate of acquisition of de novo resistance to drug *i* is *ρs*_*i*_, where *s*_*i*_ denotes the probability that de novo resistance emerges when drug *i* is used to treat an infected patient. Resistance spreads when patients infected with a resistant strain transmit it to the susceptible population.

The dynamics of this system are described mathematically using ordinary differential equations (ODE). In the following subsections we define the model for the case of one, two and three drugs and we solve analytically for the density of each compartment, which can be done because the susceptible population is held constant.

#### A single drug used in the population

In case one drug at a time is used in the population under consideration for a specific disease, the population is divided into: the Susceptibles (*S*) (being held constant) and those Infected either with susceptible strain (*W*) or a strain resistant to the drug (*X*). *W*(*t*) and *X*(*t*) denote the fraction of the population that is infected with a susceptible strain and a strain resistant to drug *X* at time *t* respectively. Infected individuals with a susceptible strain that receive treatment (*f*_1_), return to the susceptible state at rate *ρ*. Patients infected with a resistant strain (*X*) cannot be treated.

(1) $$\frac{dX\left(t\right)}{\mathrm{d}t}=\beta XS+s_{1}f_{1}\rho W-\delta X$$

(2) $$\frac{dW\left(t\right)}{\mathrm{d}t}=\beta WS-f_{1}\rho W-\delta W$$

(3) I=W+X

The solution in case of just one drug is given by the following expressions:

$$\begin{array}{c} X\left(t\right)=e^{t\left(S\beta -\delta \right)}c_{1}+\left(e^{t\left(S\beta -\delta \right)}-e^{t\left(S\beta -\delta -f_{1}\rho \right)}\right)s_{1}c_{2}\\ W\left(t\right)=e^{t\left(S\beta -\delta -f_{1}\rho \right)}c_{2} \end{array}$$

where *c*_1_ and *c*_2_ are constants determined by the initial conditions of the system.

#### Two drugs simultaneously used in the population

When two drugs are used in fractions to treat infected population (*i* = 1,2), strains resistant to either of the two drugs or to both arise and spread. The fraction of the population that is infected with a strain resistant to both drugs cannot be treated effectively. The system is described as follows:

(4) $$\frac{dX_{1}\left(t\right)}{\mathrm{d}t}=\beta X_{1}S+s_{1}f_{1}\rho W-\rho f_{2}X_{1}-\delta X_{1}$$

(5) $$\frac{dX_{2}\left(t\right)}{\mathrm{d}t}=\beta X_{2}S+s_{2}f_{2}\rho W-\rho f_{1}X_{2}-\delta X_{2}$$

(6) $$\frac{dX_{12}\left(t\right)}{\mathrm{d}t}=\beta X_{12}S+\rho \left(s_{1}f_{1}X_{2}+s_{2}f_{2}X_{1}\right)-\delta X_{12}$$

(7) $$\frac{dW\left(t\right)}{\mathrm{d}t}=\beta WS-\rho \left(f_{1}+f_{2}\right)W-\delta W$$

(8) $$I=W+X_{1}+X_{2}+X_{12}$$

Solving the system, we get the following equations for the fraction of population in each compartment at time *t*:

$$X_{1}\left(t\right)=e^{t\left(S\beta -\delta -\rho f_{2}\right)}c_{1}+\left(e^{t\left(S\beta -\delta -\rho f_{2}\right)}-e^{t\left(S\beta -\delta -\rho f_{1}-\rho f_{2}\right)}\right)c_{4}s_{1}X_{2}\left(t\right)=e^{t\left(S\beta -\delta -\rho f_{1}\right)}c_{2}+\left(e^{t\left(S\beta -\delta -\rho f_{1}\right)}-e^{t\left(S\beta -\delta -\rho f_{1}-\rho f_{2}\right)}\right)c_{4}s_{2}X_{12}\left(t\right)=e^{t\left(S\beta -\delta \right)}c_{3}+\left(e^{t\left(S\beta -\delta \right)}-e^{t\left(S\beta -\delta -\rho f_{1}\right)}\right)c_{2}s_{1}+\left(e^{t\left(S\beta -\delta \right)}-e^{t\left(S\beta -\delta -\rho f_{2}\right)}\right)c_{1}s_{2}+\left(e^{t\left(S\beta -\delta \right)}-e^{t\left(S\beta -\delta -\rho f_{1}\right)}-e^{t\left(S\beta -\delta -\rho f_{2}\right)}-e^{t\left(S\beta -\delta -\rho f_{1}-\rho f_{2}\right)}\right)c_{4}s_{1}s_{2}W\left(t\right)=e^{t\left(S\beta -\delta -\rho f_{1}-\rho f_{2}\right)}c_{4}$$

#### Three drugs simultaneously used in the population

(9) $$\frac{dX_{1}\left(t\right)}{\mathrm{d}t}=\beta X_{1}S+s_{1}f_{1}\rho W-\rho \left(f_{2}+f_{3}\right)X_{1}-\delta X_{1}$$

(10) $$\frac{dX_{2}\left(t\right)}{\mathrm{d}t}=\beta X_{2}S+s_{2}f_{2}\rho W-\rho \left(f_{1}+f_{3}\right)X_{2}-\delta X_{2}$$

(11) $$\frac{dX_{3}\left(t\right)}{\mathrm{d}t}=\beta X_{3}S+s_{3}f_{3}\rho W-\rho \left(f_{1}+f_{2}\right)X_{3}-\delta X_{3}$$

(12) $$\frac{dX_{12}\left(t\right)}{\mathrm{d}t}=\beta X_{12}S+\rho \left(s_{1}f_{1}X_{2}+s_{2}f_{2}X_{1}\right)-\rho f_{3}X_{12}-\delta X_{12}$$

(13) $$\frac{dX_{13}\left(t\right)}{\mathrm{d}t}=\beta X_{13}S+\rho \left(s_{3}f_{3}X_{1}+s_{1}f_{1}X_{3}\right)-\rho f_{2}X_{13}-\delta X_{13}$$

(14) $$\frac{dX_{23}\left(t\right)}{\mathrm{d}t}=\beta X_{23}S+\rho \left(s_{3}f_{3}X_{2}+s_{2}f_{2}X_{3}\right)-\rho f_{1}X_{23}-\delta X_{23}$$

(15) $$\frac{dX_{123}\left(t\right)}{\mathrm{d}t}=\beta X_{123}S+\rho \left(s_{1}f_{1}X_{2}3+s_{2}f_{2}X_{1}3+s_{3}f_{3}X_{1}2\right)-\delta X_{123}$$

(16) $$\frac{dW\left(t\right)}{\mathrm{d}t}=\beta WS-\rho \left(f_{1}+f_{2}+f_{3}\right)W-\delta W$$

(17) $$I=W+X_{1}+X_{2}+X_{3}+X_{12}+X_{23}+X_{13}+X_{123}$$

The proportion of the population in each compartment at time *t* is given by the following equations:

$$\begin{array}{l} X_{1}(t)=e^{t\left(S\beta -\delta -\rho f_{2}-\rho f_{3}\right)}c_{1}+(e^{t\left(S\beta -\delta -\rho f_{2}-\rho f_{3}\right)}-e^{t\left(S\beta -\delta -\rho f_{1}-\rho f_{2}-\rho f_{3}\right)}c_{8}s_{1}\\ X_{2}(t)=e^{t\left(S\beta -\delta -\rho f_{1}-\rho f_{3}\right)}c_{2}+(e^{t\left(S\beta -\delta -\rho f_{1}-\rho f_{3}\right)}-e^{t\left(S\beta -\delta -\rho f_{1}-\rho f_{2}-\rho f_{3}\right)}c_{8}s_{2}\\ X_{3}(t)=e^{t\left(S\beta -\delta -\rho f_{1}-\rho f_{2}\right)}c_{3}+(e^{t\left(S\beta -\delta -\rho f_{1}-\rho f_{2}\right)}-e^{t\left(S\beta -\delta -\rho f_{1}-\rho f_{2}-\rho f_{3}\right)}c_{8}s_{3} \end{array}$$

$$X_{12}\left(t\right)=e^{t\left(S\beta -\delta -\rho f_{3}\right)}c_{4}+\left(e^{t\left(S\beta -\delta -\rho f_{3}\right)}-e^{t\left(S\beta -\delta -\rho f_{1}-\rho f_{3}\right)}\right)c_{2}s_{1}+\left(e^{t\left(S\beta -\delta -\rho f_{3}\right)}-e^{t\left(S\beta -\delta -\rho f_{2}-\rho f_{3}\right)}\right)c_{1}s_{2}+\left(e^{t\left(S\beta -\delta -\rho f_{3}\right)}-e^{t\left(S\beta -\delta -\rho f_{1}-\rho f_{3}\right)}-e^{t\left(S\beta -\delta -\rho f_{2}-\rho f_{3}\right)}+e^{t\left(S\beta -\delta -\rho f_{1}-\rho f_{2}-\rho f_{3}\right)}\right)c_{8}s_{1}s_{2}$$

$$X_{13}\left(t\right)=e^{t\left(S\beta -\delta -\rho f_{2}\right)}c_{5}+\left(e^{t\left(S\beta -\delta -\rho f_{2}\right)}-e^{t\left(S\beta -\delta -\rho f_{1}-\rho f_{2}\right)}\right)c_{3}s_{1}+\left(e^{t\left(S\beta -\delta -\rho f_{2}\right)}-e^{t\left(S\beta -\delta -\rho f_{2}-\rho f_{3}\right)}\right)c_{1}s_{3}+\left(e^{t\left(S\beta -\delta -\rho f_{2}\right)}-e^{t\left(S\beta -\delta -\rho f_{1}-\rho f_{2}\right)}-e^{t\left(S\beta -\delta -\rho f_{2}-\rho f_{3}\right)}+e^{t\left(S\beta -\delta -\rho f_{1}-\rho f_{2}-\rho f_{3}\right)}\right)c_{8}s_{1}s_{3}$$

$$X_{23}\left(t\right)=e^{t\left(S\beta -\delta -\rho f_{1}\right)}c_{6}+\left(e^{t\left(S\beta -\delta -\rho f_{1}\right)}-e^{t\left(S\beta -\delta -\rho f_{1}-\rho f_{2}\right)}\right)c_{3}s_{2}+\left(e^{t\left(S\beta -\delta -\rho f_{1}\right)}-e^{t\left(S\beta -\delta -\rho f_{1}-\rho f_{3}\right)}\right)c_{2}s_{3}+\left(e^{t\left(S\beta -\delta -\rho f_{1}\right)}-e^{t\left(S\beta -\delta -\rho f_{1}-\rho f_{2}\right)}-e^{t\left(S\beta -\delta -\rho f_{1}-\rho f_{3}\right)}+e^{t\left(S\beta -\delta -\rho f_{1}-\rho f_{2}-\rho f_{3}\right)}\right)c_{8}s_{2}s_{3}$$

$$X_{123}\left(t\right)=e^{t\left(S\beta -\delta \right)}c_{7}+\left(e^{t\left(S\beta -\delta \right)}-e^{t\left(S\beta -\delta -\rho f_{1}\right)}\right)c_{6}s_{1}+\left(e^{t\left(S\beta -\delta \right)}-e^{t(S\beta -\delta -\rho f_{1}2}\right)c_{5}s_{2}+\left(e^{t\left(S\beta -\delta \right)}-e^{t\left(S\beta -\delta -\rho f_{3}\right)}\right)c_{4}s_{3}+\left(e^{t\left(S\beta -\delta \right)}-e^{t\left(S\beta -\delta -\rho f_{1}\right)}-e^{t\left(S\beta -\delta -\rho f_{2}\right)}+e^{t\left(S\beta -\delta -\rho f_{1}-\rho f_{2}\right)}\right)c_{3}s_{1}s_{2}+\left(e^{t\left(S\beta -\delta \right)}-e^{t\left(S\beta -\delta -\rho f_{1}\right)}-e^{t\left(S\beta -\delta -\rho f_{3}\right)}+e^{t\left(S\beta -\delta -\rho f_{1}-\rho f_{3}\right)}\right)c_{2}s_{1}s_{3}+\left(e^{t\left(S\beta -\delta \right)}-e^{t\left(S\beta -\delta -\rho f_{2}\right)}-e^{t\left(S\beta -\delta -\rho f_{3}\right)}-e^{t\left(S\beta -\delta -\rho f_{2}-\rho f_{3}\right)}\right)c_{1}s_{2}s_{3}+\left(e^{t\left(S\beta -\delta \right)}-e^{t\left(S\beta -\delta -\rho f_{1}\right)}-e^{t\left(S\beta -\delta -\rho f_{2}\right)}-e^{t\left(S\beta -\delta -\rho f_{3}\right)}+e^{t\left(S\beta -\delta -\rho f_{1}-\rho f_{2}\right)}+e^{t\left(S\beta -\delta -\rho f_{1}-\rho f_{3}\right)}+e^{t\left(S\beta -\delta -\rho f_{2}-\rho f_{3}\right)}-+e^{t\left(S\beta -\delta -\rho f_{1}-\rho f_{2}-\rho f_{3}\right)}\right)c_{8}s_{1}s_{2}s_{3}W\left(t\right)=e^{t\left(S\beta -\delta -\rho f_{1}-\rho f_{2}-\rho f_{3}\right)}c_{8}$$

The number of the differential equations needed to define the system increases exponentially in the number of drugs employed simultaneously in the population. We need to consider all possible drug combinations that the strain may acquire resistance to, in other words all the subsets of a set of cardinality *n*. Here we restrict our analysis to maximum three drugs, that we consider as a relevant number in the majority of cases. It follows directly from the above analysis how one can extend the system of equations to accommodate the case that the number of drugs used simultaneously against the same disease is larger than three.

## Footnotes

1 Global burden of disease and risk factors: Table 3A.6, p120; disability weight by disease and sequelae.
